# *Pleurotus abieticola* Polysaccharide Alleviates Hyperlipidemia Symptoms via Inhibition of Nuclear Factor-κB/Signal Transducer and Activator of Transcription 3-Mediated Inflammatory Responses

**DOI:** 10.3390/nu15234904

**Published:** 2023-11-23

**Authors:** Yongfeng Zhang, Yingjie Lin, Keyi Wu, Ming Jiang, Lanzhou Li, Yang Liu

**Affiliations:** 1 Engineering Research Center of Chinese Ministry of Education for Edible and Medicinal Fungi, Jilin Agricultural University, Changchun 130118, China; zhangyongfeng@jlau.edu.cn (Y.Z.); linyingjie@uor.edu.cn (Y.L.); wukeyi@mails.jlau.edu.cn (K.W.); 2 College of Life Science and Technology, Mudanjiang Normal University, Mudanjiang 157011, China; jiangming@mdjnu.edu.cn

**Keywords:** *Pleurotus abieticola*, hyperlipidemia, inflammation, NF-κB/STAT3 signaling pathway

## Abstract

Hyperlipidemia (HLP) is a metabolic syndrome induced by obesity, which has been widely recognized as a significant threat to human health. *Pleurotus abieticola*, an edible lignin-degrading fungus, remains relatively understudied in terms of its bioactivity and medicinal properties. In this study, the lipid-lowering effect of *Pleurotus abieticola* polysaccharide (PAPS1) was systematically explored in high-fat diet (HFD)-induced HLP mice. The findings demonstrated that the administration of PAPS1 significantly inhibited bodyweight gain, ameliorated blood glucose and lipid levels, reduced fat accumulation, and mitigated hepatic injury in HLP mice. In addition, PAPS1 demonstrated the capability to increase the levels of three distinct fecal metabolites while simultaneously reducing the levels of eight other fecal metabolites in HLP mice. According to biological detection, PAPS1 reduced the hepatic level of reactive oxygen species (ROS) and pro-inflammatory factors, such as tumor necrosis factor (TNF)-α and interleukin (IL)-1β, -6, -17A, -22, and -23, and increased the expression of anti-inflammatory factor IL-10. Combined with proteomics, Western blot and immunohistochemistry analysis showed that PAPS1 exerted suppressive effects on inflammation and oxidative damage by inhibiting the nuclear factor-κB (NF-κB)/signal transducer and activator of transcription 3 (STAT3) signaling pathway in HLP mice. These findings offer evidence supporting the effectiveness of PAPS1 as a therapeutic agent in reducing lipid levels through its targeting of chronic inflammation.

## 1. Introduction

Hyperlipidemia (HLP) is a metabolic disorder primarily induced by the consumption of a high-fat diet (HFD) or foods rich in cholesterol [1]. Its principal manifestations involve abnormal blood lipid levels, characterized by elevated total cholesterol (TC), triglyceride (TG), and low-density lipoprotein cholesterol (LDL-C), along with a diminished expression of high-density lipoprotein cholesterol (HDL-C) [2]. These dysregulated lipid profiles significantly contribute to the development of cardiovascular diseases, including atherosclerosis and coronary heart disease [1]. According to certain investigations, it is projected that approximately 40% of global fatalities will be closely linked to cardiovascular disease by the year 2030 [3]. Furthermore, there has been a significant increase in the proportion of patients diagnosed with HLP over recent decades [3]. Currently, statins are the prevailing therapeutic agents and widely recognized as the most frequently prescribed lipid-lowering drugs due to their direct impact on reducing plasma cholesterol levels, particularly LDL-C, through inhibiting cholesterol synthesis and 3-hydroxy-3-methylglutaryl-CoA reductase [4]. Nevertheless, it is worth noting that statins often exhibit adverse effects such as hepatotoxicity, nephrotoxicity, gastrointestinal disturbances, and myotoxicity [4]. Hence, there is an imperative requirement to investigate novel strategies that exhibit reduced toxicity and substantial pharmacological efficacy.

Long-term consumption of an HFD leads to the accumulation of fat in visceral organs such as the liver, kidneys, and testicles [5], which results in an increase in adipokine levels and lipid peroxidation. Ultimately, this induces systemic chronic inflammation in mice [6]. Excessive hypertrophy of white adipose tissue (WAT), serving as a significant lipid reservoir, induces the activation of hypoxic signaling to initiate oxidative stress, subsequently leading to the activation of nuclear factor-κB (NF-κB) signaling pathways [7]. NF-κB, a key transcriptional regulator in the context of inflammatory responses, induces the secretion of pro-inflammatory cytokines, including interleukin (IL)-1β and -6, and tumor necrosis factor (TNF)-α. Moreover, it facilitates the development of insulin resistance and triggers the phosphorylation of signal transducer and activator of transcription 3 (STAT3), thereby further amplifying the inflammatory response observed in obese mice [8]. During the early stage of induced differentiation in 3T3-L1 cells (a white pre-adipose cell line), there was a significant increase in phosphorylation expression of STAT3, followed by its translocation to the nucleus to mediate the transcription of adipocyte-specific genes [9]. In addition, brown adipose tissue (BAT), unlike the function of WAT, contains a large number of mitochondria, prompting fatty acids or glucose to undergo thermogenesis for the purpose of consumption [10].

The gut microbiota, considered a vital “organ” of the host organism, plays a crucial role in lipid metabolism and immunomodulation processes [11]. The gut microbiota has the ability to impact cholesterol metabolism, lipid peroxidation, and intestinal barrier permeability. Therefore, it plays a significant role in the development and progression of HLP [12]. Recently, natural polysaccharides derived from mushrooms have demonstrated their ability to regulate intestinal flora and its metabolites, thereby offering a theoretical foundation for our research on their potential in reducing blood lipids.

Natural compounds derived from mushrooms have garnered increasing attention in recent years due to their rich physiological activities, health benefits, and few side-effects [13]. Modern pharmacological studies have demonstrated that polysaccharides derived from mushrooms exhibit a diverse range of pharmacological activities, encompassing immunomodulatory, hypoglycemic, anti-inflammatory, antioxidant, hypolipidemic, hepatoprotective, and anticancer properties [13]. Jiang et al. found that polysaccharides derived from *Grifola frondosa* (GFPA) effectively reduced body weight and blood glucose levels, mitigated HLP, and suppressed inflammation through the Toll-like receptor 4 (TLR4)/NF-κB signaling pathway in HFD-fed mice [14]. Numerous studies have demonstrated the beneficial impact of *Pleurotus* fungal polysaccharides, specifically those derived from *Pleurotus eryngii*, *Pleurotus nebrodensis*, and *Pleurotus ostreatus*, on lipid reduction in blood [15,16,17]. *Pleurotus abieticola* (*P. abieticola*), a fungus known for its ability to degrade lignin and used as an edible species, is found in northeastern and northwestern regions of China, as well as the Russian Far East [18]. It has been successfully cultivated on *Pinus massoniana* chips administered by nematodes [19]. Recently, we successfully isolated a neutral polysaccharide (PAPS1) from *P. abieticola* that possessed a well-defined structure and exhibited remarkable efficacy in modulating the gut microflora, activating the Nrf2 signaling pathway, suppressing oxidative stress, and enhancing the immune response in cyclophosphamide-induced immunosuppressed mice [18]. However, the potential of PAPS1 to mitigate hyperlipidemic symptoms by modulating fecal metabolite levels remains unexplored.

As the pharmacological mechanisms of *P. abieticola* and its polysaccharides have received limited attention, especially regarding obesity and hyperlipidemia, further research is necessary to investigate their potential therapeutic effects in these conditions. In this study, we developed a mouse model of HLP induced by HFD to investigate the hypolipidemic efficacy and potential mechanism of PAPS1 treatment. Combined with the analysis of intestinal flora metabolomics and hepatic proteomics, PAPS1 was found to exert its inhibitory effect on the inflammatory response by modulating the NF-κB/STAT3 signaling pathway, thereby effectively ameliorating symptoms associated with HLP. These results support the use of PAPS1 as a hypolipidemic agent that targets chronic inflammation.

## 2. Materials and Methods

### 2.1. Chemicals

PAPS1 was sequentially isolated and purified from an aqueous extract of the *P. abieticola* fruiting bodies using hot-water extraction, protein removal, ethanol precipitation, anion exchange chromatography, and gel filtration chromatography according to our previous research. It had a molecular weight of 17.16 kDa and consisted of a main backbone containing →6)-α-D-Galp-(1→, →2,6)-α-D-Galp-(1→ and →3)-β-D-Glcp-(1→ residues. Additionally, the branch was substituted at the C-2 position of →2,6)-α-D-Galp-(1→ residue by a β-D-Manp-(1→ and β-D-Manp-(1→6)-α-D-Galp-(1→ residue [18]. HFD (Cat: D12492) was purchased from the Xiao Shu You Tai Biotechnol`ogy Co., Ltd. (Beijing, China). Normal chow diet (NCD, Cat: D12450B) was obtained from the Changsheng Biotechnology Co., Ltd. (Benxi, China). Simvastatin (SV, Lot: H20093943) was purchased from Hengrui Pharmaceutical Co., Ltd. (Chengdu, China). Blood glucose meter and test strips were obtained from sinocare (Changsha, China). Radioimmunoprecipitation assay (RIPA) buffer (20-188) and electrochemiluminescence immunoassay (ECL) kit (WBKLS0500) were obtained from Merck Millipore (Darmstadt, Germany). Protease and phosphatase inhibitors (P002) and rapid closure solution (P30500) were obtained from New Cell & Molecular Biotech (Suzhou, China). Sodium dodecyl sulfate-polyacrylamide gel electrophoresis (SDS-PAGE) (10–12%; PG112) was obtained from EpiZyme (Shanghai, China). Polyvinylidene fluoride (PVDF) membranes (0.45 μm; 10600023) were obtained from Cytiva (Marlborough, MA, USA). Details regarding biochemical assay and enzyme-linked immunosorbent assay (ELISA) kits are provided in Table S1. Detailed information regarding the antibodies employed in immunohistochemistry and Western blot techniques is elucidated in Table S2.

### 2.2. Establishment of Animal Model

The experimental protocols were approved by the Institutional Ethics Committee of Jilin Agricultural University (approval No. 20220926001). A total of 32 specific pathogen-free male C57BL/6J mice, aged 5 weeks, were procured from Changsheng Biotechnology Co., Ltd. The experimental animals were maintained under standard conditions with a room temperature of 23 ± 1 °C, humidity ranging between 40 and 60%, and a light/dark cycle of 12 h each. Ample food and water were provided ad libitum to ensure their well-being.

**Figure 1:**
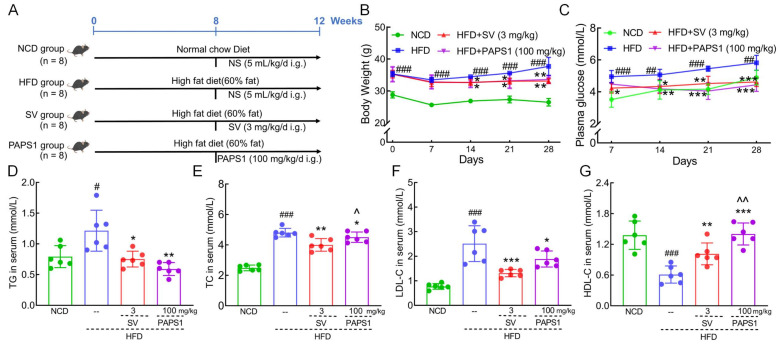
PAPS1 ameliorated hyperlipidemia symptoms in HFD-induced HLP mice. (**A**) The HLP model establishment and administration process. PAPS1 treatment suppressed the body weight (**B**) and plasma glucose (**C**) gain; decreased the serum levels of TG (**D**), TC (**E**), and LDL-C (**F**); and increased the serum level of HDL-C (**G**) in HFD-induced HLP mice. Data are expressed as the mean ± S.E.M. (n = 6). # *p* < 0.05, ## *p* < 0.01, and ### *p* < 0.001 vs. NCD group; * *p* < 0.05, ** *p* < 0.01, and *** *p* < 0.001 vs. HFD group; ^ *p* < 0.05 and ^^ *p* < 0.01 vs. SV group. NCD: normal chow diet; HFD: high-fat diet; SV: simvastatin; PAPS1: *Pleurotus abieticola* polysaccharide; TG: triglyceride; TC: total cholesterol; LDL-C: low-density lipoprotein cholesterol; HDL-C: high-density lipoprotein cholesterol.

The HLP model establishment and administration process are shown in Figure 1A. Twelve mice were randomly selected to be fed an NCD (detailed ingredients are provided in Table S3), while the remaining mice were fed an HFD (detailed ingredients are provided in Table S3) to establish an HLP model for a duration of 12 weeks, as illustrated in Figure 1A. After eight weeks of nourishment, the HLP model was randomly partitioned into three groups (n = 8) based on their body weight, including the HFD group (model), SV group (positive, 3 mg/kg), and PAPS1 group (100 mg/kg). During the subsequent 4-week period, mice in the NCD and HFD groups were orally administered normal saline (NS) at a dosage of 5 mL/kg, while mice in the SV and PAPS1 groups intragastrically received their respective drugs on a daily basis. Furthermore, throughout the experimental duration, the body weight and fasting plasma glucose levels of each group of mice were meticulously monitored and documented on a weekly basis. After the last administration, the mice underwent an 8 h fasting period followed by euthanasia using CO_2_ for blood collection, as well as retrieval of cecum faces, organs (liver, spleen, kidney, heart, lung, muscle, pancreas, and thymus), WAT (epididymal (eWAT), perirenal (pWAT), and inguinal (iWAT)), and scapula BAT.

### 2.3. Organ Index

The organs and adipose tissues mentioned above were monitored for organ index using the following formula: organ index (%) = (weight of organ (g)/body weight (g)) × 100. The organ index of the NCD group was established as the reference values for assessing the organ index of HFD group, SV group, and PAPS1 group.

### 2.4. Histopathological Analysis

Parts of the aforementioned organs and adipose tissues were fixed in 4% paraformaldehyde for a duration of 48 h. Subsequently, they underwent paraffin embedding, sectioning at a thickness of 5 μm, dewaxing, and dehydration to obtain slides. Then, histopathological examination of the above organs and adipose tissues was conducted using hematoxylin and eosin (H&E) staining as well as Oil red O staining, following the methodology employed in our previous study [20].

### 2.5. Immunohistochemical Staining (IHC)

The deparaffinized liver and adipose tissue slides from the NCD, HFD, and PAPS1 groups were subjected to a 10 min incubation in 3% H_2_O_2_ to block non-specific antibody binding. Subsequently, primary antibodies (cellular repressor of E1A-stimulated genes 1 (Creg1), 4-hydroxynonenal (4-HNE), P-NF-κB) and HRP-labeled secondary antibodies (Table S2) were applied followed by hematoxylin counterstaining for imaging using an optical microscope (BX51; Olympus, Tokyo, Japan), as previously described [21].

### 2.6. Detection of Biochemical Indicators

Doses of 2.5 μL of serum from all groups of mice were subjected to biochemical assays, following the manufacturer’s instructions, to determine the levels of TC, TG, LDL-C, and HDL-C in the serum. The liver tissues of all groups were homogenized in saline at ratio of 1:10 (*w*/*v*), followed by centrifugation to collect the resulting liver supernatant. Subsequently, 5 μL doses of liver supernatant were analyzed for protein content using a bicinchoninic acid (BCA) kit. Then, 10 μL doses of serum and/or liver supernatant from all groups were assayed for aspartate transaminase (AST), alanine aminotransferase (ALT), and reactive oxygen species (ROS) contents using ELISA as per the operating instructions provided in the manual. Additionally, 100 μL doses of liver supernatant were assayed for IL-1β, IL-6, IL-10, IL-17A, IL-22, IL-23, and TNF-α levels using uncoated ELISA kits according to the operating instructions in the manual (Table S1).

### 2.7. Metabolomics Analysis

The fecal samples from the cecum of the NCD, HFD, and PAPS1 groups (n = 4) were subjected to vacuum freeze-drying. Subsequently, they were weighed, homogenized, and crushed before being centrifuged to obtain the supernatant. Then, the supernatant was analyzed and data processed using ultra-high performance liquid chromatography-tandem mass spectrometry (UPLC-MS/MS)-based non-targeted metabolomics, as previously described [21,22].

### 2.8. Proteomic Analysis

The liver tissue samples from the NCD, HFD, and PAPS1 groups were homogenized and lysed using RIPA buffer. Subsequently, acetone precipitation was performed followed by resuspension, trypsin digestion, desalting, and finally vacuum freeze-drying to obtain peptides. Then, peptides were subsequently subjected to UPLC-MS/MS analysis, employing label-free quantification (LFQ) and MaxQuant (2.0.1.0; Max Planck Institute of Bio chemistry), as previously described [23,24].

### 2.9. Western Blot (WB)

The liver tissue from all groups was homogenized and lysed using ice-cold high-efficiency RIPA buffer supplemented with 1% protease and phosphatase inhibitors. Subsequently, the samples were centrifuged twice at 14,000× *g* for 5 min at 4 °C to obtain protein supernatants. Then, denatured protein (40 μg) was separated using a 10–12% SDS-PAGE gel and transferred onto PVDF membranes. The membranes were then blocked using a rapid closure solution and incubated with primary and secondary antibodies (Table S1). Finally, the protein bands were visualized using an ECL kit and analyzed using the Tanon 5200 automated chemiluminescence image analysis system (Tanon, Shanghai, China). The densitometry analysis of protein bands was conducted utilizing ImageJ software (v1.8.0; National Institutes of Health, Bethesda, MD, USA), with comparison to β-actin as the standard protein.

### 2.10. Statistical Analysis

All values are presented as means ± S.E.M. Biochemical indices were compared between different groups using one-way analysis of variance (ANOVA) followed by Dunnett’s *t*-test using BONC DSS Statistics 25 (IBM, Armonk, NY, USA). Differences were considered statistically significant at *p* < 0.05.

## 3. Results

### 3.1. PAPS1 Inhibited Bodyweight Gain, Alleviated Dysglycemia and Dyslipidemia, and Decreased Fat Accumulation in HLP Mice

To investigate the anti-hyperlipidemic effect of PAPS1, parameters of HLP in HFD-fed mice according to the experimental arrangement are outlined in Figure 1A. Compared to NCD mice, the HFD intake for 12 weeks resulted in a significant increase in body weight gain (*p* < 0.001, Figure 1B) and plasma glucose levels (*p* < 0.01, Figure 1C) in mice. However, the administration of 100 mg/kg PAPS1 and 3 mg/kg SV over a period of 4 weeks resulted in a reduction in these enhancements. The body weight and plasma glucose levels had no significant difference between PAPS1- and SV-treated mice (*p* > 0.05, Figure 1B,C). In addition, long-term consumption of HFD resulted in significant increases in serum levels of TG (*p* < 0.05, Figure 1D), TC (*p* < 0.001, Figure 1E), and LDL-C (*p* < 0.001, Figure 1F), while simultaneously decreasing the level of HDL-C (*p* < 0.001, Figure 1G) compared to the NCD group. Treatment with PAPS1 and SV for 4 weeks exhibited a significant reduction in TG levels (*p* < 0.05, Figure 1D), TC levels (*p* < 0.05, Figure 1E), and LDL-C levels (*p* < 0.05, Figure 1F), along with a notable elevation in HDL-C levels (*p* < 0.01, Figure 1G) in HFD-induced HLP mice. Compared with SV-treated mice, PAPS1 had a weaker effect on reducing TC levels (*p* < 0.05, Figure 1E) and a stronger effect on increasing HDL-C levels (*p* < 0.01, Figure 1G), with no difference on TG and LDL-C (*p* > 0.05, Figure 1D,F).

**Figure 2:**
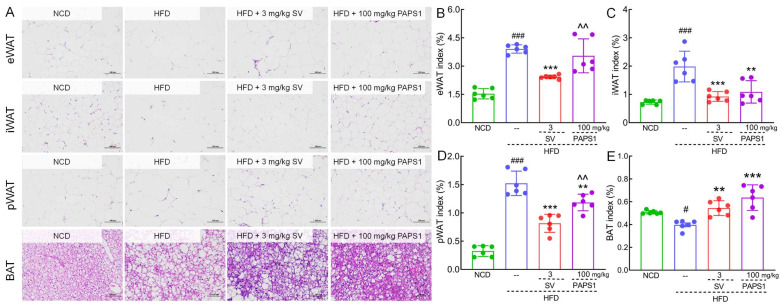
PAPS1 inhibited fat accumulation in HFD-induced HLP mice. (**A**) Histopathological analysis of eWAT, iWAT, pWAT, and BAT was performed using H&E staining (200×; scale bar: 100 μm) (n = 3). PAPS1 treatment had no significant effect on eWAT index (**B**) but reduced the indexes of iWAT (**C**) and pWAT (**D**) and enhanced the BAT index (**E**) in HFD-induced HLP mice. Data are expressed as the mean ± S.E.M. (n = 6). # *p* < 0.05 and ### *p* < 0.001 vs. NCD group; ** *p* < 0.01 and *** *p* < 0.001 vs. HFD group; ^^ *p* < 0.01 vs. SV group. NCD: normal chow diet; HFD: high-fat diet; SV: simvastatin; PAPS1: *Pleurotus abieticola* polysaccharide; eWAT: epididymal white adipose tissue; iWAT: inguinal white adipose tissue; pWAT: perirenal white adipose tissue; BAT: brown adipose tissue.

Fat accumulation in the body is commonly considered a prominent marker for obesity and HLP [25]. As expected, histological analyses of adipose tissue showed that the adipocyte size of WAT and BAT in HLP mice was remarkably large, but the number was obviously less, compared to those of NCD mice (Figure 2A). Interestingly, treatment with PAPS1 and SV for a duration of 4 weeks significantly suppressed adipocyte hypertrophy and promoted an increase in the number of adipocytes, effectively mitigating hyperplasia in HLP mice (Figure 2A). These findings suggest that PAPS1 possesses the ability to inhibit excessive fat accumulation in HLP mice. In addition, as shown in Figure 2B–E, the indexes of eWAT (*p* < 0.001), iWAT (*p* < 0.001), and pWAT (*p* < 0.001) were increased, and the index of BAT (*p* < 0.05) was decreased in HLP mice in comparison to the NCD mice. However, PAPS1 intervention significantly reversed the aforementioned indices except for eWAT index, whereas SV intervention notably reversed these indices. Compared with SV-treated mice, PAPS1 had a weaker effect on reducing the eWAT index and pWAT index (*p* < 0.01, Figure 2B,D). Overall, our findings indicated that PAPS1 ameliorated systemic excessive lipid accumulation in HFD-induced HLP mice.

### 3.2. PAPS1 Attenuated Hepatic Injury in HLP Mice

**Figure 3:**
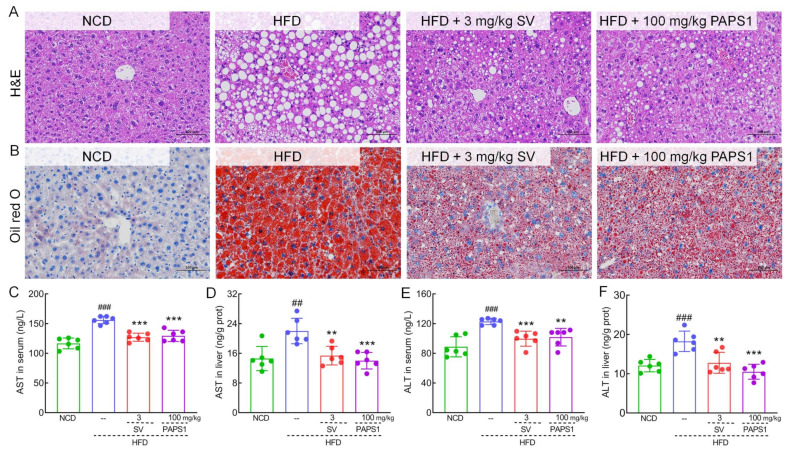
PAPS1 improved liver injury in HFD-induced HLP mice. Histopathological analysis of liver was performed using H&E staining (**A**) and oil red O staining (**B**) (200×; scale bar: 100 μm) (n = 3). PAPS1 treatment reduced the levels of AST (**C**,**D**) and ALT (**E**,**F**) in serum and liver of HLP mice caused by HFD intake. Data are expressed as the mean ± S.E.M. (n = 6). ## *p* < 0.01 and ### *p* < 0.001 vs. NCD group; ** *p* < 0.01 and *** *p* < 0.001 vs. HFD group. NCD: normal chow diet; HFD: high-fat diet; SV: simvastatin; PAPS1: *Pleurotus abieticola* polysaccharide; H&E: hematoxylin and eosin; ALT: alanine aminotransferase; AST: aspartate transaminase.

As shown in Figure 3A,B, chronic HFD intake induced severe hepatic steatosis, lipid vacuole accumulation, swelling, as well as inflammatory cell infiltration in the liver of HLP mice, which were significantly alleviated through PAPS1 and SV intervention for 4 weeks. Similarly, compared with NCD mice, HFD promoted an elevated liver index of HLP mice (*p* < 0.01, Table S4), but PAPS1 treatment for 4 weeks could effectively reduce the liver index (*p* < 0.05, Table S4). As biomarkers of liver injury, the serum and liver levels of AST (*p* < 0.01, Figure 3C,D) and ALT (*p* < 0.001, Figure 3E,F) of HLP mice were significantly higher than those of the NCD group, indicating that the HFD caused severe liver damage in mice. However, 4-week PAPS1 and SV intervention reduced the contents of AST (*p* < 0.01, Figure 3C,D) and ALT (*p* < 0.01, Figure 3E,F) of the serum and liver in HLP mice. There was no significant difference in liver index, AST, and ALT levels between the PAPS1- and SV-treated groups. Thus, these results suggested that PAPS1 improved HFD-induced hepatic damage.

Furthermore, we investigated the pathological effect and organ indexes of PAPS1 on other organs. The result of H&E staining showed no obvious pathological changes in the kidney, spleen, heart, lung, thymus, and muscle among all groups (Figure S1). Interestingly, long-term HFD intake significantly reduced the indexes of the heart (*p* < 0.05) and pancreas (*p* < 0.001) in mice, and PAPS1 intervention obviously enhanced the indexes of the thymus (*p* < 0.05) and pancreas (*p* < 0.01) in HLP mice (Table S4). Apart from the indexes of the heart, thymus, and pancreas, there were no significant differences in the indexes of the kidney, spleen, and lung among all groups (Table S4).

### 3.3. PAPS1 Regulated Fecal Metabolites in HLP Mice

**Figure 4:**
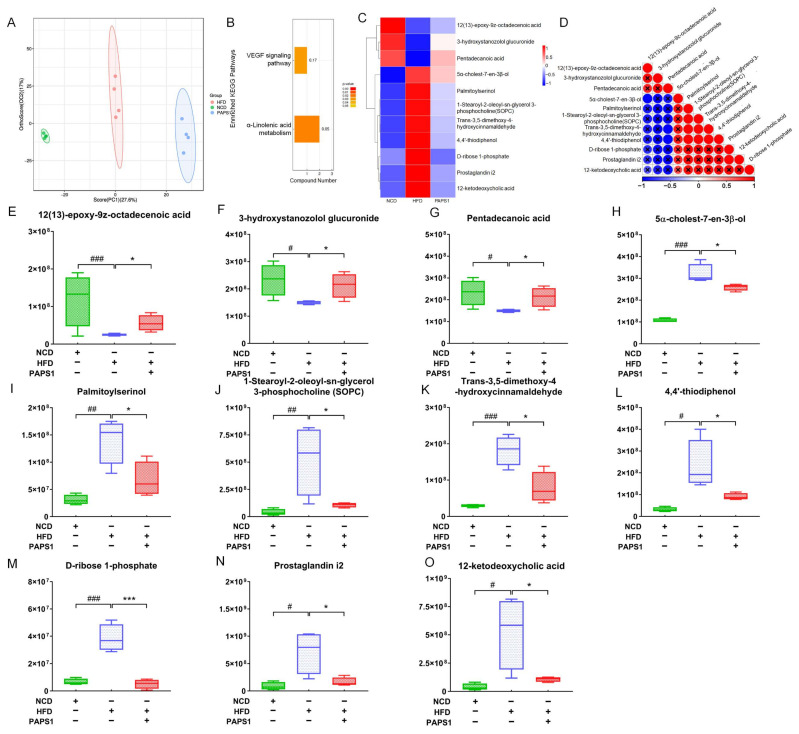
PAPS1 regulated the fecal metabolite level in HFD-induced HLP mice. (**A**) OPLS-DA score plot of fecal metabolites among NCD, HFD, and PAPS1 groups. (**B**) KEGG-enriched metabolic pathways of significantly altered fecal metabolites between HFD and PAPS1 groups. Heatmap (**C**), the associated heatmap (**D**), and boxplots (**E**–**O**) of significantly differential metabolites in intestinal feces among three groups. Data are expressed as the mean ± S.E.M. (n = 4). # *p* < 0.05, ## *p* < 0.01, and ### *p* < 0.001 vs. NCD group; * *p* < 0.05 and *** *p* < 0.001 vs. HFD group. KEGG: Kyoto Encyclopedia of Genes and Genomes; VEGF: vascular endothelial growth factor; NCD: normal chow diet; HFD: high-fat diet; PAPS1: *Pleurotus abieticola* polysaccharide.

To further clarify whether PAPS1 intervention improved dyslipidemia and its relationship with changes in fecal metabolites in HLP mice, non-targeted metabolomics was employed to explore fecal samples from NCD, HFD, and PAPS1 groups. The analysis of these samples identified a total of 1058 metabolites, including 477 positive ion and 581 negative ion metabolites. According to the results of orthogonal partial least-squares discriminant analysis (OPLS-DA), significant differences were observed among three groups, suggesting that chronic HFD intake and PAPS1 intervention induced remarkable changes at fecal metabolic levels of mice (Figure 4A). Based on the variable importance in projection (VIP) scores > 1 and *p*-value < 0.05 of OPLS-DA, a total of 189 significantly differential fecal metabolites (79 up-regulated and 110 down-regulated) were observed in the comparison between HFD and NCD groups (Figures S2 and S3), and 43 significantly differential fecal metabolites (26 up-regulated and 17 down-regulated) were observed in the comparison between PAPS1 and HFD groups (Figures S4 and S5). Kyoto Encyclopedia of Genes and Genomes (KEGG) pathway enrichment analysis of the PAPS1 and HFD groups revealed that significantly differential metabolites were mainly involved in the alpha-linolenic acid metabolism and VEGF signaling pathway (Figure 4B). In addition, after a comparison of significantly differential metabolites among the three groups, the results revealed that compared to the NCD group, chronic HFD intake obviously down-regulated the levels of 12(13)-epoxy-9z-octadecenoic acid (*p* < 0.001, Figure 4E), 3-hydroxystanozolol glucuronide (*p* < 0.05, Figure 4F), and pentadecanoic acid (*p* < 0.05, Figure 4G), and up-regulated the levels of 5α-cholest-7-en-3β-ol (*p* < 0.001, Figure 4H), palmitoyl serinol (*p* < 0.01, Figure 4I), 1-stearoyl-2-oleoyl-sn-glycerol 3-phosphocholine (SOPC) (*p* < 0.01, Figure 4J), trans-3,5-dimethoxy-4- hydroxycinnamaldehyde (*p* < 0.001, Figure 4K), 4,4′-thiodiphenol (*p* < 0.05, Figure 4L), D-ribose 1-phosphate (*p* < 0.001, Figure 4M), prostaglandin i2 (*p* < 0.05, Figure 4N), and 12-ketodeoxycholic acid (*p* < 0.05, Figure 4O) in fecal samples of mice, which were reversed through PAPS1 intervention for 4 weeks (Figure 4C). A significant negative correlation was found between the levels of pentadecanoic acid and those of 12-ketodeoxycholic acid (Figure 4D). Overall, these results demonstrate that the intervention of PAPS1 effectively modulates the aberrant expression of metabolites in fecal samples induced by prolonged HFD.

### 3.4. Proteomics Analysis in Liver of HLP Mice

**Figure 5:**
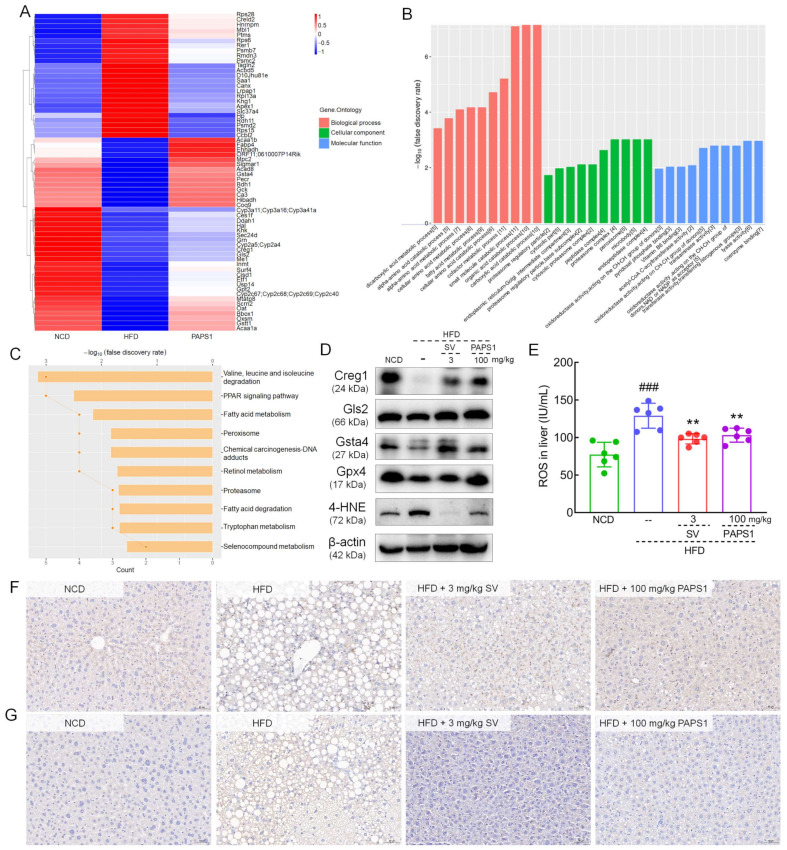
Proteomics of analysis of the liver in HFD-induced HLP mice. Heatmap (**A**), GO (**B**), and KEGG (**C**) enrichment analysis of significantly altered proteins among NCD, HFD, and PAPS1 groups. (**D**) The WB results showed PAPS1 treatment increased hepatic levels of Creg1, Gls2, Gsta4, and Gpx4 and decreased the hepatic level of 4-HNE in HFD-induced HLP mice. (**E**) PAPS1 treatment reduced the hepatic level of ROS in HFD-induced HLP mice. Data are expressed as the mean ± S.E.M. (n = 6). ### *p* < 0.001 vs. NCD group; ** *p* < 0.01 vs. HFD group. The IHC analysis showed PAPS1 treatment enhanced the hepatic expression of Creg1 (**F**) and reduced the expression of 4-HNE (**G**) in HFD-induced HLP mice. NCD: normal chow diet; HFD: high-fat diet; SV: simvastatin; PAPS1: *Pleurotus abieticola* polysaccharide; Creg1: cellular repressor of E1A-stimulated genes 1; Gls2: glutaminase 2; Gsta4: glutathione S-transferase alpha 4; Gpx4: glutathione peroxidase 4; 4-HNE: 4-hydroxynonenal.

To further elucidate the mechanisms underlying and the key proteins of PAPS1 on HFD-induced HLP mice, LFQ was performed to explore proteomic changes in the liver among the three groups. Based on the ratio A/B ≥ 1.5 and unique peptide ≥ 2 of protein expression levels, a total of 530 differential proteins were identified between NCD and HFD groups, while 169 differential proteins were identified between HFD and PAPS1 groups. Among these, chronic HFD intake increased the expression of 25 types of protein and decreased the expression of 39 types of protein in the liver of HLP mice, which were attenuated through PAPS1 intervention for 4 weeks (Figure 5A and Table S5). Detailed results of gene ontology (GO) enrichment analysis including the biological process, cellular compartment, and molecular function are depicted in Figure 5B and Figure S6A–C. PAPS1 intervention mainly affected the fatty acid metabolic process, oxidoreductase activity (acting on the CH-CH and CH-OH groups of donors, and NAD or NADP as acceptors), and peroxisome. KEGG pathway enrichment analysis showed that the differential proteins were mapped to several pathways, such as valine, leucine and isoleucine degradation; the PPAR signaling pathway; fatty acid metabolism; peroxisome; chemical carcinogenesis-DNA adducts; retinol metabolism; proteasome; and fatty acid degradation (Figure 5C). Overall, these results suggested that PAPS1 treatment regulated liver-associated proteins to promote fatty acid metabolism and oxidoreductase activity in HFD-induced HLP mice.

Based on the differential expression protein assessment, Creg1, glutaminase 2 (Gls2), and glutathione S-transferase alpha 4 (Gsta4) were further analyzed using WB or IHC. Long-term HFD intake suppressed the hepatic levels of Creg1 (*p* < 0.001, Figure 5D and Figure S7A), Gls2 (*p* < 0.001, Figure 5D and Figure S7B), Gsta4 (*p* < 0.001, Figure 5D and Figure S7C), and glutathione peroxidase 4 (Gpx4) (*p* < 0.001, Figure 5D and Figure S7D), and enhanced the expression of 4-HNE (*p* < 0.001, Figure 5D and Figure S7E) in the liver of mice. Comparatively, the 4-week PAPS1 administration significantly increased the level of Creg1 (*p* < 0.001, Figure 5D and Figure S7A), Gls2 (*p* < 0.001, Figure 5D and Figure S7B), Gsta4 (*p* < 0.001, Figure 5D and Figure S7C), and Gpx4 (*p* < 0.001, Figure 5D and Figure S7D), and inhibited the expression of 4-HNE (*p* < 0.001, Figure 5D and Figure S7E) in the liver of HFD-induced HLP mice. However, the SV intervention notably reversed these expressions except for Gpx4, and the Gsta4 increase effect and 4-HNE decrease effect of PAPS1 were weaker than those of SV (*p* < 0.01, Figure 5D and Figure S7C,E). In addition, in HFD-induced HLP mice, a high level of ROS was noted in the liver (*p* < 0.001, Figure 5E), which was significantly reversed through PAPS1 and SV intervention (*p* < 0.01, Figure 5E). Similar to the results of WB, the IHC analysis showed that PAPS1 and SV treatment strongly up-regulated the contents of Creg1 (*p* < 0.05, Figure 5F and Figure S8A) and down-regulated the level of 4-HNE (*p* < 0.01, Figure 5G and Figure S8B) in the liver of HFD-induced HLP mice. Overall, these results suggested that PAPS1 treatment could increase the levels of antioxidant-related proteins and inhibit the expression of 4-HNE and ROS to ameliorate lipid peroxidation and suppress oxidative stress in HFD-induced HLP mice.

### 3.5. PAPS1 Modulated NF-κB/STAT3 Signaling Pathway to Inhibit Inflammation in HLP Mice

**Figure 6:**
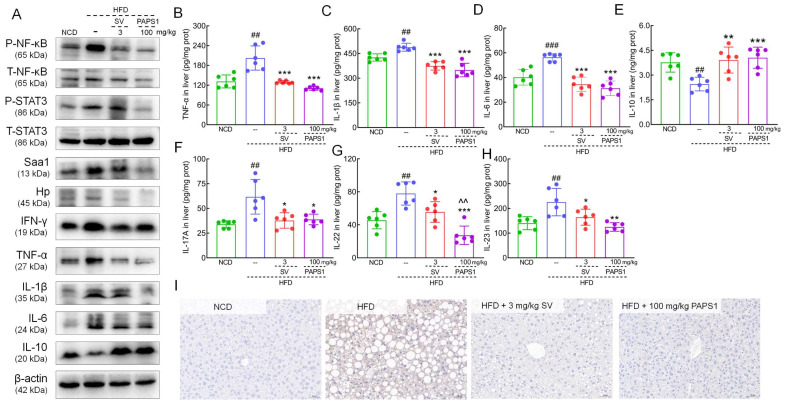
PAPS1 treatment inhibited NF-κB/STAT3-mediated inflammatory response in HFD-induced HLP mice. (**A**) PAPS1 treatment suppressed NF-κB/STAT3 signaling pathway and its related downstream proteins of liver in HFD-induced HLP mice. PAPS1 treatment down-regulated the hepatic levels of TNF-α (**B**), IL-1β (**C**), IL-6 (**D**), IL-17A (**F**), IL-22 (**G**), and IL-23 (**H**), and up-regulated the hepatic level of IL-10 (**E**) in HFD-induced HLP mice. Data are expressed as the mean ± S.E.M. (n = 6). ## *p* < 0.01 and ### *p* < 0.001 vs. NCD group; * *p* < 0.05, ** *p* < 0.01, and *** *p* < 0.001 vs. HFD group; ^^ *p* < 0.01 vs. SV group. (**I**) PAPS1 treatment suppressed the hepatic level of P-NF-κB in HFD-induced HLP mice, as analyzed using IHC. NF-κB: nuclear factor-κB; P-NF-κB: phosphorylated NF-κB; STAT3: signal transducer and activator of transcription 3; P-STAT3: phosphorylated STAT3; Saa1: serum amyloid A protein; Hp: haptoglobin; IFN-γ: interferon-γ; TNF-α: tumor necrosis factor; IL-1β: interleukin-1β; HFD: high-fat diet; NCD: normal chow diet; PAPS1: *Pleurotus abieticola* polysaccharide; SV: simvastatin.

The acute inflammatory response factor of proteomics analysis, namely, serum amyloid A protein (Saa1) and Haptoglobin (Hp), provided a clear direction to further study the potential hypolipidemic effect of PAPS1 associated with inflammation. NF-κB is a typical pro-inflammatory signaling pathway that, when activated, promotes the release of inflammatory factors [26]. As shown in Figure 6A, PAPS1 significantly reduced the hepatic expressions of P-NF-κB (*p* < 0.001, Figure S7F), P-STAT3 (*p* < 0.001, Figure S7G), Saa1 (*p* < 0.001, Figure S7H), Hp (*p* < 0.001, Figure S7I), interferon-γ (IFN-γ) (*p* < 0.001, Figure S7J), TNF-α (*p* < 0.001, Figure S7K), IL-1β (*p* < 0.001, Figure S7L), and IL-6 (*p* < 0.001, Figure S7M), and increased the level of IL-10 (*p* < 0.001, Figure S7N) in HFD-induced HLP mice. Similar to PAPS1 treatment, SV intervention notably reversed these proteins except for P-NF-κB and Saa1 in HFD-induced HLP mice. In addition, the results of ELISA showed that PAPS1 intervention resulted in a decrease in the levels of TNF-α, IL-1β, IL-6, IL-17A, IL-22, and IL-23 by >45.3% (*p* < 0.001; Figure 6B), >28.4% (*p* < 0.001; Figure 6C), >44.5% (*p* < 0.005; Figure 6D), >37.0% (*p* < 0.05; Figure 6F), >65.0% (*p* < 0.001; Figure 6G), and >44.6% (*p* < 0.01; Figure 6H), respectively, and in an increase in the levels of IL-10 by >65.2% (*p* < 0.001; Figure 6E) in the liver of HFD-induced HLP mice. Compared with SV-treated mice, PAPS1 had a stronger effect on reducing IL-22 levels (*p* < 0.01, Figure 6G). The suppressive effect of PAPS1 on the level of phosphorylated NF-κB in the liver of HFD-induced HLP mice was further confirmed through the IHC analysis (*p* < 0.01; Figure 6I and Figure S8C). These results indicated that PAPS1 treatment inhibited the NF-κB/STAT3 signaling pathway to alleviate inflammation in HFD-induced HLP mice.

## 4. Discussion

In this study, the ameliorative impact of PAPS1 on HLP was confirmed through assessments of bodyweight, organ indexes, histopathological staining, and biochemical assays in HLP mice. PAPS1 intervention effectively ameliorated the HLP phenotype by inducing reductions in body weight and plasma glucose levels, as well as restoring disturbances in serum lipid metabolism (down-regulating TC, TG, and LDL-C and up-regulating HDL-C). These preliminary findings suggested that PAPS1 possessed the potential to serve as a therapeutic agent for ameliorating symptoms related to hyperlipidemia. Adipose tissue plays a crucial role in maintaining the overall energy balance of the body and undergoes dynamic remodeling to align with the nutritional requirements of the organism [27]. The abnormal remodeling of adipose tissue in obesity is primarily characterized by the hypertrophy of adipocytes, inadequate lipogenesis, increased fibrosis, and infiltration of immune cells [27]. PAPS1 intervention effectively augmented adipocyte proliferation and concurrently inhibited adipocyte hypertrophy in both white and brown adipose tissue, thereby facilitating the remodeling of adipose tissue in HFD-induced HLP mice.

The excessive intake of HFD disrupts the delicate balance of gut microbiota, resulting in an increase in intestinal permeability [28]. This disruption facilitates the entry of microbiota metabolites, particularly short-chain fatty acids and lipopolysaccharides (LPSs), into the entero-hepatic circulation. Ultimately, this process contributes to the development of chronic inflammation associated with obesity or HLP [29]. Thus, in order to specifically demonstrate the impact of PAPS1 on the fecal metabolism of gut microbiota in HLP mice, this study identified that PAPS1 significantly modulated the levels of 43 fecal metabolites and was involved in the metabolism of α-linolenic acid as well as the VEGF signaling pathway within the intestinal feces of HLP mice, as determined through an untargeted metabolomics investigation. α-linolenic acid, a plant-derived essential fatty acid, is primarily metabolized into energy and CO_2_ through β-oxidation [30]. Additionally, a minor portion undergoes conversion into eicosapentaenoic acid and docosahexaenoic acid via the action of desaturases and elongase-2 [30]. These metabolic processes contribute to its anti-metabolic syndrome effects, including anti-obesity and anti-hyperlipidemia properties, as well as improvements in cardiovascular health [30]. The activation of the VEGF signaling pathway enhances the expression levels of UCP1 and proliferator-activated receptor-co-activator 1-α, thereby inducing WAT browning and promoting brown/beige adipogenesis. Consequently, this leads to an increase in energy expenditure and a beneficial improvement in metabolism [31]. PAPS1 intervention effectively reversed the abnormal levels of 11 metabolites in fecal samples of mice induced by chronic HFD intake. Pentadecanoic acid, being a long-chain saturated fatty acid, has demonstrated effective anti-inflammatory and antioxidant properties, particularly in overweight adolescents. Furthermore, it exhibits a significant inverse association with TC levels [32]. Muhammad et al. discovered a significant increase in serum levels of 12-ketodeoxycholic acid, a crucial component involved in the absorption of dietary fat and maintenance of cholesterol balance, among mice induced with a high-fat protein diet [33]. This finding aligns with our fecal metabolite results obtained from mice subjected to an HFD-induced condition. PAPS1 significantly elevated the levels of pentadecanoic acid and reduced the levels of 12-ketodeoxycholic acid in fecal samples from HLP mice. Furthermore, a significant negative correlation was observed between pentadecane acid and 12-ketodeoxycholic acid. Therefore, PAPS1 exhibited the ability to modulate fecal metabolites, thereby enhancing lipid metabolism in HLP mice.

Hepatic lipid metabolism plays a crucial role in obesity and HLP [34]. Here, we performed a detailed proteomic analysis of the liver to explore the potential mechanism of PAPS1 on HFD-induced HLP mice. PAPS1 treatment could regulate the PPAR signaling pathway, fatty acid metabolism, peroxisome, fatty acid degradation, and oxidoreductase activity, which were all involved in regulating de novo lipogenesis and promoting lipolysis. Gls2, a crucial enzyme involved in the conversion of glutamine to glutamate, plays a vital role in the synthesis of GSH and clearance of ROS, thereby ensuring the functional integrity of mitochondria [35]. However, its expression is significantly diminished in CCl_4_-induced NASH mice [36] as well as ApoE^−/−^ mice fed with a Western diet [37]. Excess ROS initiate the lipid peroxidation of polyunsaturated fatty acids, forming a large number of toxic electrophilic substances and free radicals, leading to a significant increase in 4-HNE content and inducing oxidative damage in liver tissues [38]. 4-HNE, a highly prevalent aldehyde found in lipid peroxidation byproducts, significantly contributes to signaling pathways triggered by oxidative stress and the detrimental effects of oxidant toxicity [38]. Gsta4 is a detoxifying enzyme that promotes the metabolism of 4-HNE through binding to GSH [39]. It has been found that altered oxidative stress and inflammatory responses in adipose tissue caused by obesity are closely related to the down-regulation of Gsta4 [40]. The antioxidant Gpx4 effectively hinders the production of ROS caused by oxidative stress in adipose tissue, thereby mitigating adipose inflammation through the prevention of lipid peroxidation in obesity [41]. PAPS1 intervention increased the expression of Gls2, Gsta4, and Gpx4, and reduced the levels of ROS and 4-HNE to inhibit lipid peroxidation in HFD-induced HLP mice.

Overexpression of Creg1, a glycoprotein secreted in mice and humans, up-regulated the expression of brown-fat-related genes, and promoted the brown adipogenesis in murine mesenchymal stem cell line C3H10T1/2 [42]. But the expression of *Creg1* mRNA was obviously reduced during white adipogenesis in 3T3-L1 cells after differentiation stimulation [42]. PAPS1 intervention improved the hepatic Creg1 level and increased the BAT index in HFD-induced HLP mice. Interestingly, Creg haploinsufficiency in Creg^+/−^ heterozygous mice amplified HFD-induced inflammation, as evidenced by the increased cytokine expression such as TNF-α, IL-6, and MCP-1 via activation of the NF-κB signaling pathway [43].

Obesity can lead to the development of chronic inflammation, characterized by a systemic and persistent low-grade inflammatory response triggered by diverse inflammatory factors [44]. Hp, a glycoprotein implicated in the hepatic response to acute inflammation, is regarded as a biomarker for obesity and inflammatory conditions [45]. Hp mRNA expression was markedly increased in the WAT of obese leptin-receptor-deficient db/db mice, while Hp mRNA expression showed a significant down-regulation in TNF-α-deficient ob/ob mice [46]. Hp plays a crucial role in initiating adipocyte inflammation during the progression of obesity, and its expression is detected early on in adipocytes during the onset of obesity and further increases with weight gain [45]. We found that the Hp level in the liver of HFD mice increased and was down-regulated after administration of PAPS1. NF-κB plays an important role in regulating liver inflammation [47], and STAT3 is another important transcription factor involved in immune response and inflammation, which can interact with NF-κB and cooperate to control inflammation [48]. Thus, upon activation of the NF-κB/STAT3 signaling pathway, it triggers the activation of diverse immune cells including macrophages, neutrophils, and T cells, as well as the production of inflammatory cytokines such as IL-1β, IL-6, and TNF-α [49]. The activation of the NF-κB pathway triggers the secretion of IL-6, subsequently leading to STAT3 deactivation and the enhanced expression of STAT3 target genes [50]. IL-10 serves as a pivotal anti-inflammatory factor, while STAT3 assumes a critical function in the IL-10-mediated anti-inflammatory response [51]. STAT3 exhibits pro-inflammatory effects, whereas upon stimulation by IL-10, STAT3 demonstrates anti-inflammatory properties [51]. Our study found that the expression level of IL-10 significantly increased after administration of PAPS1. In addition, long-term HFD intake resulted in a significant increase in Saa1 protein levels in the liver of mice [52]. Saa1, a hepatic stress protein with pro-inflammatory properties, triggers immune cell activation and endothelial cell destruction, thereby facilitating the recruitment of inflammatory factors and platelets [53]. This ultimately contributes to the progression of diseases associated with metabolic syndrome [53]. Previous studies have demonstrated that Saa1 exerts a stimulatory effect on the intracellular and extracellular translocation of the NF-κB p65 protein, thereby indicating its potential to activate the NF-κB signaling pathway [52]. Saa1 establishes a regulatory circuit involving Saa1/TLR4/NF-κB/Saa1 feedback, which serves as a trigger for the development of liver steatosis and the intrahepatic inflammatory response [54]. In our study, the administration of PAPS1 resulted in a down-regulation in phosphorylation levels of NF-κB and STAT3, as well as Saa1 and pro-inflammatory cytokines including IFN-γ, TNF-α, IL-1β, and IL-6 in the liver of HFD-induced HLP mice. These findings suggest that PAPS1 may mitigate inflammation induced by a high-fat diet through modulation of the NF-κB/STAT3 signaling pathway.

There were some limitations in our study. Firstly, the structural characteristics of the polysaccharide facilitate a comprehensive exploration of its mechanism of action; however, despite the preliminary characterization of PAPS1’s structure in previous studies, further analysis is required to elucidate the intricate relationship between its structure and antihyperlipidemic efficacy. Secondly, our findings have demonstrated that PAPS1 exerts a regulatory influence on fecal metabolite levels in mice with HFD-induced HLP, potentially through alterations in the composition of the gut microbiota. Consequently, further investigations are warranted to delve into the intricate interplay between fecal metabolites and gut microbiota. Finally, PAPS1 intervention effectively promoted adipose tissue remodeling in HFD-induced HLP mice, potentially associated with enhanced insulin sensitivity. Therefore, further analysis is required to elucidate the mechanism underlying the relationship between adipose tissue remodeling and insulin resistance.

## 5. Conclusions

In summary, *Pleurotus abieticola* polysaccharide (PAPS1) exhibited the potential to enhance intestinal metabolism and mitigated inflammation and oxidative damage, thereby alleviating HLP symptoms in HFD-induced mice. Additionally, it regulated lipid metabolism through the NF-κB/STAT3 signaling pathway. The findings of this study will serve as a valuable research foundation for the development of lipid-lowering medications.

## Data Availability

Data are contained within the article and Supplementary Materials.

## Supplementary Materials

The following supporting information can be downloaded at https://www.mdpi.com/article/10.3390/nu15234904/s1. Figure S1: Histopathological analysis of the organs (kidney, spleen, heart, lung, thymus, and muscle) of mice using H&E staining (200×; scale bar: 100 μm). NCD: normal chow diet; HFD: high-fat diet; SV: simvastatin; PAPS1: *Pleurotus abieticola* polysaccharide. Figure S2: Differential fold analysis of the significantly differential fecal metabolites in the negative ion modes between NCD and HFD groups. Figure S3: Differential fold analysis of the significantly differential fecal metabolites in the positive ion modes between NCD and HFD groups. Figure S4: Differential fold analysis of significantly different metabolites in the negative ion modes between HFD and PAPS1 groups. Figure S5: Differential fold analysis of significantly different metabolites in the positive ion modes between HFD and PAPS1 groups. Figure S6: Pie chart of biological process (**A**), cellular component (**B**), and molecular function (**C**) classification of GO enrichment analysis. Figure S7: Quantification of protein expression in Figure 5D and Figure 6A normalized to that of β-actin and expressed as the percentage of NCD group (n = 3). The data are shown as the mean ± S.E.M. ### *p* < 0.001 vs. NCD group; *** *p* < 0.001 vs. HFD group; ^^ *p* < 0.01 and ^^^ *p* < 0.001 vs. SV group. NCD: normal chow diet; HFD: high-fat diet; SV: simvastatin; PAPS1: *Pleurotus abieticola* polysaccharide; Creg1: cellular repressor of E1A-stimulated genes 1; Gls2: glutaminase 2; Gsta4: glutathione S-transferase alpha 4; Gpx4: glutathione peroxidase 4; 4-HNE: 4-hydroxynonenal; P-NF-κB: phosphorylated NF-κB; P-STAT3: phosphorylated STAT3; Saa1: serum amyloid A protein; Hp: haptoglobin; IFN-γ: interferon-γ; TNF-α: tumor necrosis factor-α; IL-1β: interleukin-1β. Figure S8: Quantification of protein expression in Figure 5E–G and Figure 6I normalized to that of β-actin and expressed as the percentage of NCD mice (n = 3). The data are shown as the mean ± S.E.M. # *p* < 0.05, ## *p* < 0.01, and ### *p* < 0.001 vs. NCD group; * *p* < 0.05, ** *p* < 0.01, and *** *p* < 0.001 vs. HFD group. NCD: normal chow diet; HFD: high-fat diet; SV: simvastatin; PAPS1: *Pleurotus abieticola* polysaccharide; Creg1: cellular repressor of E1A-stimulated genes 1; 4-HNE: 4-hydroxynonenal; P-NF-κB: phosphorylated NF-κB. Table S1: Details of kits used in biochemical assay and ELISA. Table S2: Details of antibodies used in IHC and WB. Table S3: Formula of normal chow diet and high-fat diet. Table S4: The effect PAPS1 on organ indexes in HLP mice. Table S5: Proteins with significantly differential expression levels in proteomics.
