# Supplementary material for: Pleurotus abieticola Polysaccharide Alleviates Hyperlipidemia Symptoms via Inhibition of Nuclear Factor-κB/Signal Transducer and Activator of Transcription 3-Mediated Inflammatory Responses
Source: Nutrients. 2023 Nov 23;15(23):4904. doi: 10.3390/nu15234904 (PMC10708251; doi:10.3390/nu15234904)
Supplement: Supplementary file 1 [file nutrients-15-04904-s001.zip › nutrients-2732430-supplementary.pdf]

Pleurotus abieticola Polysaccharide Alleviates Hyper-lipidemia Symptoms via Inhibition of Nuclear Factor-κB/Signal Transducer and Activator of Transcription 3-Mediated Inflammatory Responses

Yongfeng Zhang[1], Yingjie Lin[1], Keyi Wu[1] Ming Jiang[2], Lanzhou Li[1*], Yang Liu[1*]

[1] Engineering Research Center of Chinese Ministry of Education for Edible and Medicinal Fungi, Jilin Agricultural University, Changchun, 130118, China
[2] College of Life Science and Technology, Mudanjiang Normal University, Mudanjiang, 157011, China.

*Corresponding authors:

Yang Liu, Ph.D.
Engineering Research Center of Chinese Ministry of Education for Edible and Medicinal Fungi, Jilin Agricultural University,
Changchun, Jilin, P.R.China.
Email: y_liu10@jlau.edu.cn

Lanzhou Li, Ph.D.
Engineering Research Center of Chinese Ministry of Education for Edible and Medicinal Fungi, Jilin Agricultural University,
Changchun, Jilin, P.R.China.
Email: lilanzhou@jlau.edu.cn

Yongfeng Zhang, Email: zhangyongfeng@jlau.edu.cn
Yingjie Lin, Email: linyingjie@uor.edu.cn
Keyi Wu, Email: wukeyi@mails.jlau.edu.cn
Ming Jiang, Email: jiangming@mdjnu.edu.cn

Supplementary data

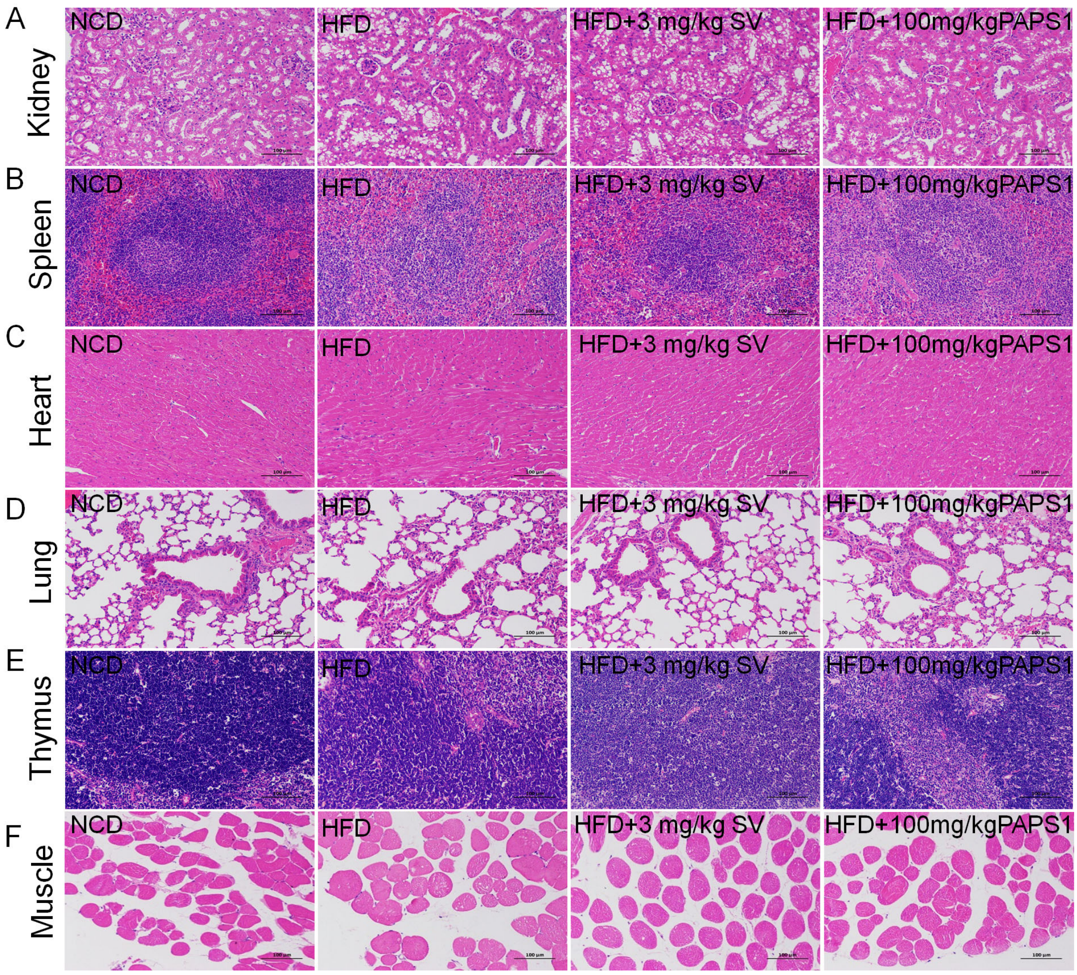

Figure S1. Histopathological analysis of the organs (kidney, spleen, heart, lung, thymus, and muscle) of mice using H&E staining (200×; scale bar: 100 μm). NCD: normal chow diet; HFD: high-fat diet; SV: simvastatin; PAPS1: *Pleurotus abieticola* polysaccharide.

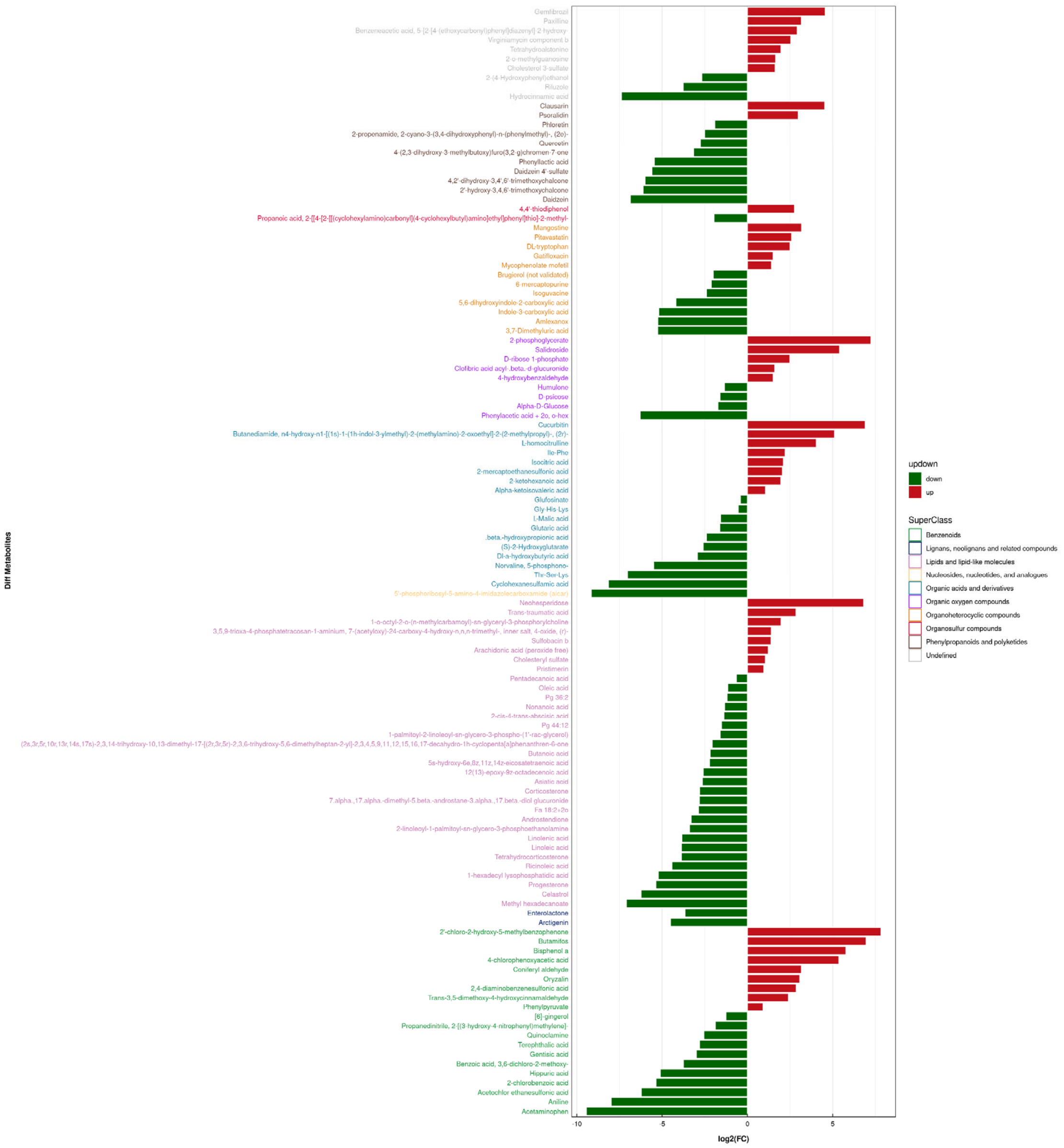

Figure S2. Differential fold analysis of the significantly differential fecal metabolites in the negative ion modes between NCD and HFD group.

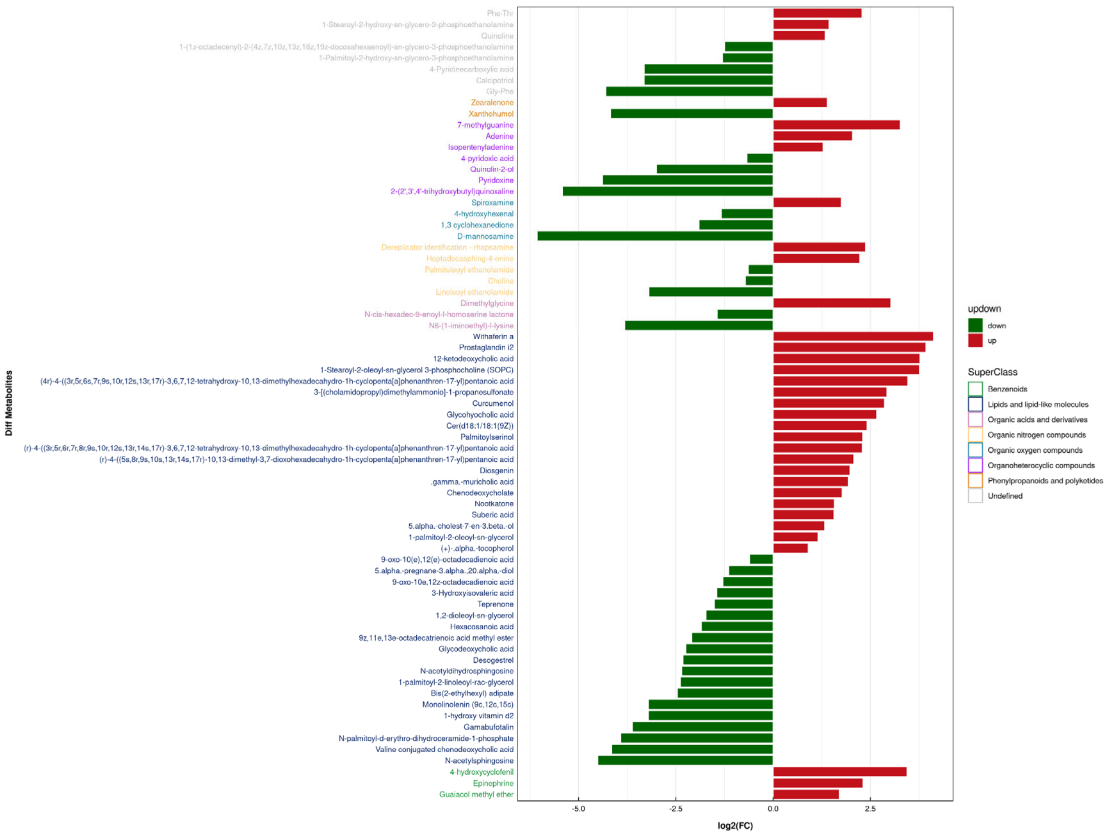

Figure S3. Differential fold analysis of the significantly differential fecal metabolites in the positive ion modes between NCD and HFD group.

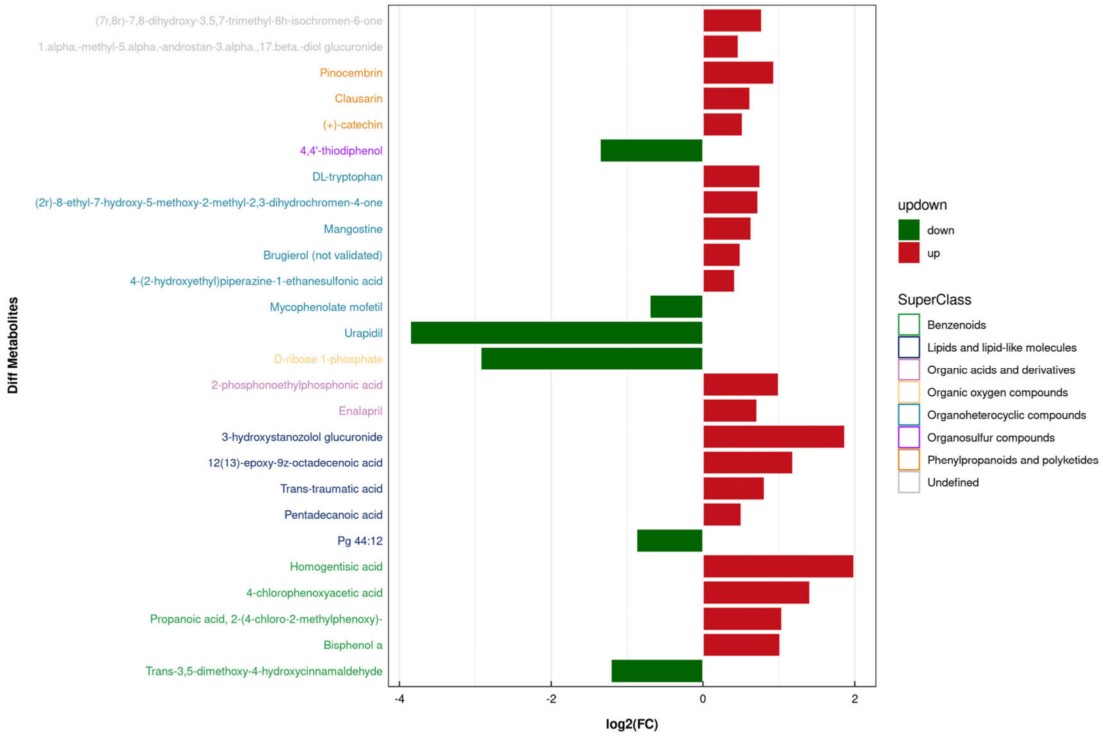

Figure S4. Differential fold analysis of significantly different metabolites in the negative ion modes between HFD and PAPS1 group.

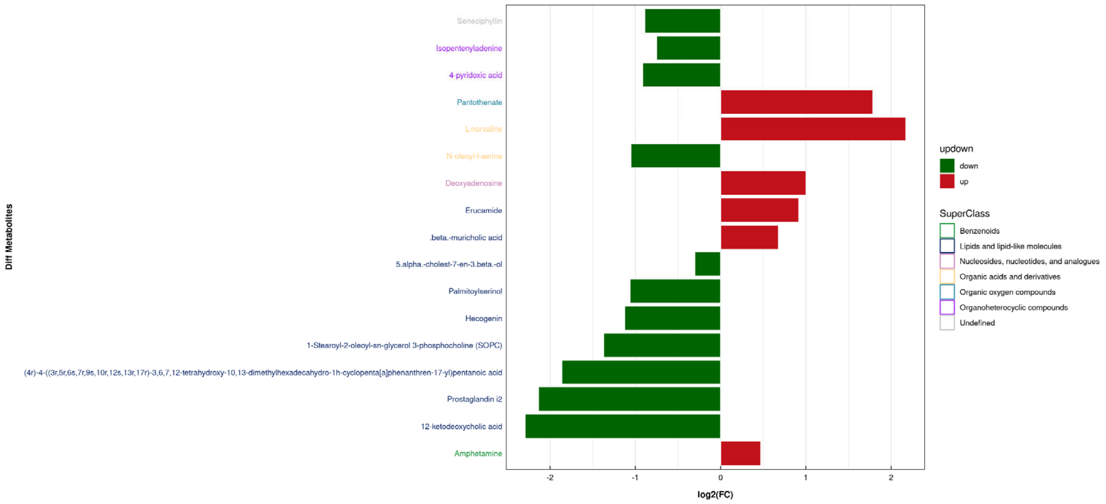

Figure S5. Differential fold analysis of significantly different metabolites in the positive ion modes between HFD and PAPS1 group.

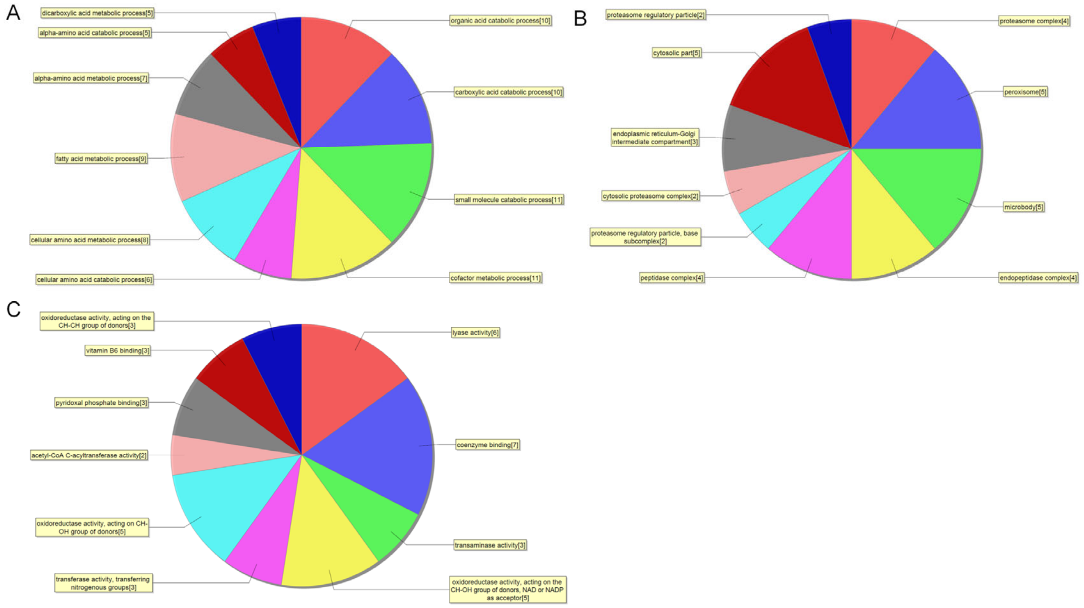

Figure S6. Pie chart of biological process **(A)**, cellular component **(B)**, molecular function **(C)** classification of GO enrichment analysis.

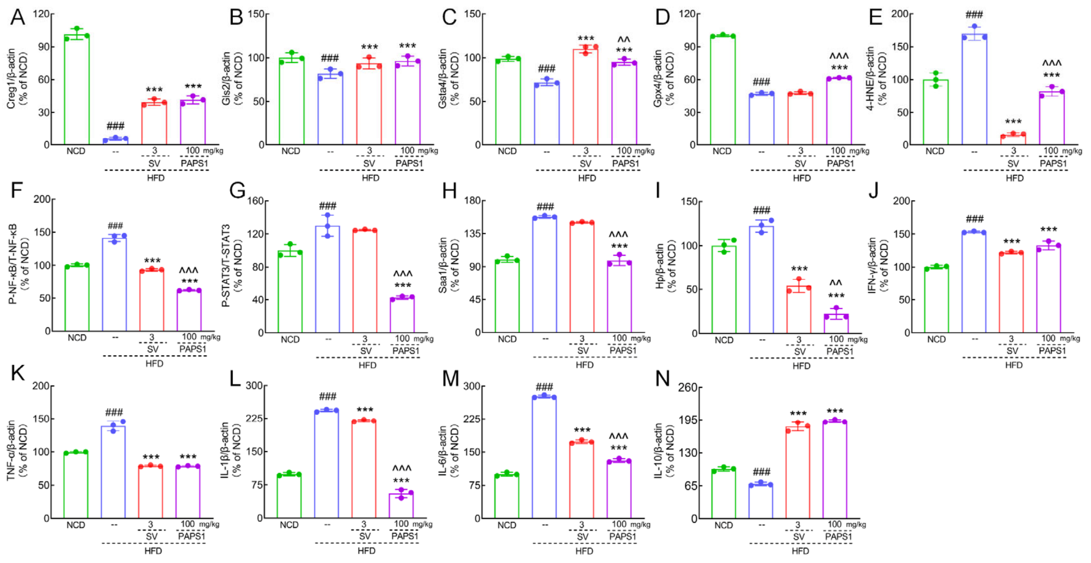

Figure S7. Quantification of protein expression in Figure 5D and 6A normalized to that of β-actin and expressed as the percentage of NCD group (n = 3). The data are shown as the mean ± S.E.M. ###$p < 0.001$ versus NCD group; ***$p < 0.001$ versus HFD group; ^^ $p < 0.01$, and ^^^ $p < 0.001$ versus SV group. NCD: normal chow diet; HFD: high-fat diet; SV: simvastatin; PAPS1: *Pleurotus abieticola* polysaccharide; Creg1: cellular repressor of E1A-stimulated genes 1; Gls2: glutaminase 2; Gsta4: glutathione S-transferase alpha 4; Gpx4: glutathione peroxidase 4; 4-HNE: 4-hydroxynonenal.; P-NF-κB: phosphorylated NF-κB; P-STAT3: phosphorylated STAT3; Saa1: serum amyloid A protein; Hp: haptoglobin; IFN-γ: interferon-γ; TNF-α: tumor necrosis factor-α; IL-1β: interleukin-1β.

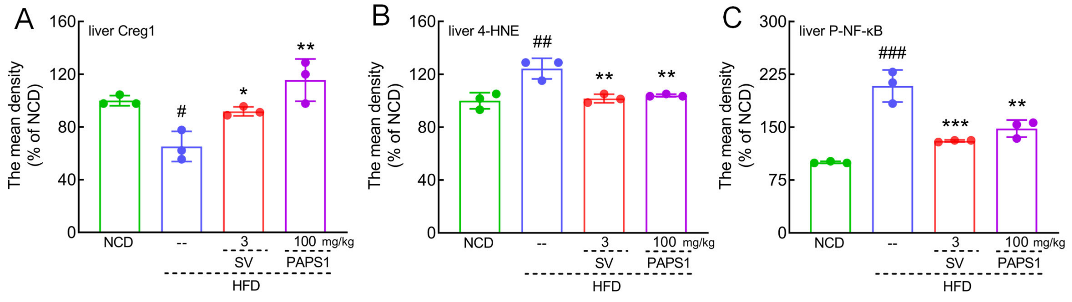

Figure S8. Quantification of protein expression in Figure 5E-G and 6I normalized to that of β-actin and expressed as the percentage of NCD mice (n = 3). The data are shown as the mean ± S.E.M. #$p < 0.05$, ##$p < 0.01$ and ###$p < 0.001$ versus NCD group; *$p < 0.05$, **$p < 0.01$ and ***$p < 0.001$ versus HFD group. NCD: normal chow diet; HFD: high-fat diet; SV: simvastatin; PAPS1: *Pleurotus abieticola* polysaccharide; Creg1: cellular repressor of E1A-stimulated genes 1; 4-HNE: 4-hydroxynonenal; P-NF-κB: phosphorylated NF-κB.

Table S1. Details of kits used in biochemical assay and ELISA.

Kits	Catalog number	Company	Area
TC detection kit	A111-1-1	Nanjing Jiancheng Bioengineering Institute	Nanjing, China
TC detection kit	A110-1-1	Nanjing Jiancheng Bioengineering Institute	Nanjing, China
LDL-C detection kit	A113-1-1	Nanjing Jiancheng Bioengineering Institute	Nanjing, China
HDL-C detection kit	A112-1-1	Nanjing Jiancheng Bioengineering Institute	Nanjing, China
Mouse ALT ELISA kit	MM-44625M1	Meimian Biotechnology	Yancheng, China
Mouse AST ELISA kit	MM-44115M1	Meimian Biotechnology	Yancheng, China
Mouse ROS ELISA kit	MM-43700M1	Meimian Biotechnology	Yancheng, China
Mouse TNF-α uncoated ELISA kit	88-7324	Thermo Fisher Scientific	MA, USA
Mouse IL-1β uncoated ELISA kit	88-7013	Thermo Fisher Scientific	MA, USA
Mouse IL-6 uncoated ELISA kit	88-7064	Thermo Fisher Scientific	MA, USA
Mouse IL-10 uncoated ELISA kit	88-7105	Thermo Fisher Scientific	MA, USA
Mouse IL-17A uncoated ELISA kit	88-7371	Thermo Fisher Scientific	MA, USA
Mouse IL-22 uncoated ELISA kit	88-7422	Thermo Fisher Scientific	MA, USA
Mouse IL-23 uncoated ELISA kit	88-7230	Thermo Fisher Scientific	MA, USA
BCA kit	23225	Thermo Fisher Scientific	MA, USA

Table S2. Details of antibodies used in IHC and WB

Antibody	Molecular weight	Catalog number	Dilution for WB	Dilution for IHC	Company	Area
P-NF-κB	65 kDa	AF2006	1:1000	1:500	Affinity	Jiangsu, China
T-NF-κB	65 kDa	A18210	1:2000	/	Abclonal	Wuhan, China
P-STAT3	86 kDa	AF3293	1:2000	/	Affinity	Jiangsu, China
T-STAT3	92 kDa	3096489	1:2000	/	MILLIPORE	Shanghai, China
IL-1β	17/35 kDa	A16288	1:2000	/	Abclonal	Wuhan, China
IL-6	23 kDa	bs-0782R	1:2000	/	Bioss	Beijing,China
TNF-α	17/25 kDa	A0277	1:2000	/	Abclonal	Wuhan, China
IL-10	20/22 kDa	A2171	1:1000	/	Abclonal	Wuhan, China
IFN-γ	19 kDa	DF6045	1:2000	/	Affinity	Jiangsu, China
Creg1	24 kDa	DF10194	1:3000	1:500	Affinity	Jiangsu, China
Gsta4	27 kDa	DF12203	1:2000	/	Affinity	Jiangsu, China
Gls2	66 kDa	DF13386	1:2000	/	Affinity	Jiangsu, China
Hp	45 kDa	381731	1:1000	/	ZEN BIO	Chengdu, China
Saa1	13 kDa	ER62519	1:2000	/	HuaBio	Hangzhou, China
Gpx4	17 kDa	A19544	1:2000	/	Abclonal	Wuhan, China
4-HNE	72 kDa	ab46545	1:2000	1:500	Abcam	Cambridge, UK
β-actin	43 kDa	Sc-47778	1:2000	/	Santa Cruz	TX, USA
Goat Anti-Rabbit (H+L)	/	E-AB-1003	1:2000	1:400	Elabscience	TX, USA
Goat-Anti-Mouse (H+L)	/	E-AB-1001	1:2000	1:400	Elabscience	TX, USA

Table S3. Formula of normal chow diet and high-fat diet.

	NCD	HFD
Fat (kcal%)	10%	60%
Protein (kcal%)	20%	20%
Carbohydrate (kcal%)	70%	20%
Total (kcal%)	100%	100%

NCD: normal chow diet; HFD: high-fat diet; SV: simvastatin; PAPS1: *Pleurotus abieticola* polysaccharide.

Table S4. The effect PAPS1 on organ indexes in HLP mice.

	Organ	NCD	HFD		
				3 mg/kg SV	100 mg/kg PAPS1
Organ index（%）	Liver	3.929±0.139	4.586±0.077##	4.119±0.143	4.105±0.037*
	Kidney	1.451±0.032	1.425±0.017	1.475±0.041	1.473±0.038
	Spleen	0.337±0.012	0.333±0.024	0.362±0.026	0.336±0.018
	Heart	0.703±0.018	0.631±0.016#	0.699±0.005*	0.665±0.008
	Lung	0.606±0.086	0.58±0.017	0.586±0.014	0.572±0.015
	Thymus	0.070±0.003	0.052±0.003	0.065±0.004	0.073±0.007*
	Pancreas	0.796±0.029	0.590±0.017###	0.704±0.015*	0.737±0.026**

The data were analyzed using a one-way ANOVA and expressed as means ± S.E.M. (n = 6). #p < 0.05, ##p < 0.01 and ###p < 0.001 *versus* NCD group, *p < 0.05 and **p < 0.01 *versus* HFD group. NCD: normal chow diet; HFD: high-fat diet; SV: simvastatin; PAPS1: *Pleurotus abieticola* polysaccharide.

Table S5. Proteins with significantly differential expression levels in proteomics

Number	Protein. names	Unique. peptides	fc. NCD-HFD	fc. HFD-PAPS1
Up-regulated proteins by PAPS1 (Number:39)				
1	Surf4	2	39.118139	0.0465283
2	Fabp4	4	7.4147893	0.0712626
3	Cyp3a11;Cyp3a16;Cyp3a41a	6	75.610755	0.1147315
4	Oxsm	2	7.0736367	0.1739606

5	Gsta4	2	4.4557578	0.232885
6	Scrn2	2	5.5890002	0.2454149
7	Bbox1	2	3.8376455	0.2995994
8	Acaa1b	5	2.0669158	0.3618472
9	Sigmar1	2	2.4689319	0.3670756
10	Etf1	3	4.6934167	0.3737625
11	Inmt	11	4.2251969	0.3767676
12	ORF11;0610007P14Rik	2	1.8730031	0.3849625
13	Grn	2	7.6496016	0.3984767
14	Ca3	16	2.3182793	0.4141417
15	Creg1	2	5.9268832	0.4161873
16	Cyp2a5;Cyp2a4	5	5.5953594	0.4324899
17	Ehhadh	30	1.5761045	0.4836167
18	Mtatp8	2	2.6396857	0.498133
19	Ddah1	7	3.7453863	0.5263316
20	Mpc2	4	1.6294373	0.5414203
21	Sec24d	2	4.4387162	0.5575168
22	Coq9	3	1.7423806	0.5597148
23	Oat	11	1.9601638	0.5867929
24	Usp14	2	2.480496	0.5873441
25	Acaa1a	4	1.8526392	0.593132
26	Khk	11	2.9074653	0.6037921
27	Ces1f	15	2.658276	0.6038403
28	Me1	13	3.0997432	0.6125076
29	Acad8	8	1.6742251	0.6209792
30	Bdh1	9	1.598835	0.6212225
31	Gck	5	1.57656	0.6235898
32	Hibadh	8	1.5479204	0.6323804
33	Cisd1	4	2.2395791	0.6401977
34	Cyp2c67;Cyp2c68;Cyp2c69;Cyp2c40	7	2.1551439	0.6405674
35	Gstt1	8	1.6869598	0.6471396
36	Hal	14	2.5095091	0.6494651
37	Pecr	5	1.5320279	0.6512129
38	Gls2	8	2.7497557	0.6537813
39	Gpt2	5	2.0618183	0.6592659
Down-regulated proteins by PAPS1 (Number:25)				
1	Ptms	2	0.2024234	1.5057212
2	Kng1	3	0.6039607	1.5063507
3	Mbl1	2	0.1350671	1.5108965
4	Hnrnpm	5	0.2355895	1.5144546
5	Lrpap1	2	0.6098233	1.5453759

6	Creld2	3	0.1830977	1.5608068
7	Rer1	2	0.3462425	1.5711873
8	Rpl13a	5	0.5687837	1.5767496
9	Slc37a4	2	0.5201448	1.6010325
10	Psmb7	2	0.2572866	1.619552
11	Psmc2	3	0.2652712	1.6283054
12	Rdh11	2	0.655161	1.6459288
13	Rps28	2	0.1257974	1.6765039
14	Canx	15	0.5321497	1.734502
15	Rmdn3	3	0.1884984	1.735627
16	Apex1	2	0.4296374	1.7507134
17	Rps6	5	0.317078	1.7868004
18	Psmd2	8	0.6314325	1.8031546
19	D10Jhu81e	7	0.5121806	1.8408467
20	Ccbl2	6	0.6094626	1.8646155
21	Acbd5	3	0.3950492	2.4316103
22	Hp	11	0.546026	3.1897325
23	Saa1	2	0.1559159	4.0626199
24	Tagln2	2	0.2171844	4.3434152
25	Rps15	2	0.3475577	4.4990631

fc. NCD-HFD: the ratio of protein between NCD group and HFD group;

fc. HFD-PAPS1: the ratio of protein between HFD group and PAPS1 group.

NCD: normal chow diet; HFD: high-fat diet; PAPS1: *Pleurotus abieticola* polysaccharide.
